# Rhino-Orbital-Cerebral Mucormycosis and Orbital Exenteration

**Published:** 2016-11-02

**Authors:** Matthew P. Fahrenkopf, Joshua J. Nelson, Mitchell Eichhorn, Julie Conway, Adam Hassan

**Affiliations:** ^a^Michigan State University College of Human Medicine, Grand Rapids; ^b^Grand Rapids Medical Education Partners Plastic and Reconstructive Surgery Residency, Grand Rapids, Mich; ^c^Helen DeVos Children's Hospital Pediatric Ophthalmology, Grand Rapids, Mich; ^d^Eye Plastic & Facial Cosmetic Surgery, Grand Rapids, Mich

**Keywords:** mucormycosis, fungus, orbit, exenteration, diabetes

## DESCRIPTION

A 43-year-old man presented to the emergency department with diabetic ketoacidosis, left facial pain, and swelling. Black eschar was noted to be protruding from both external nasal cavities. A computed tomographic (CT) scan was obtained, demonstrating sinus wall thickening and decreased attenuation of the left temporal lobe. Air fluid levels were also visualized in the sphenoid, left maxillary, and left ethmoid sinuses.

## QUESTIONS

1. **What is mucormycosis?**
2. **Are there specific associated risk factors?**
3. **What are common presenting signs and symptoms?**
4. **What are treatment options for rhino-orbital-cerebral mucormycosis?**

## DISCUSSION

Mucormycosis is a rare fungal infection, occurring mostly in immunocompromised patients, which can be potentially deadly. It is caused by the zygomycete, Mucorales, which are ubiquitous saprophytic fungi found in the soil and decomposing organic matter.^1^^,^^2^ These fungi are classically characterized by nonseptate, wide ribbon-shaped hyphae, branching at right angles ranging from 45° to 90°. Mucormycosis has a high affinity for arteries, allowing it to disseminate throughout the body via hematologic channels. This secondarily can lead to thrombosis and ischemia of surrounding tissues.^3^ Infection by this fungal species starts via host inhalation and then spreads to other areas of the body.

Immunocompromised patients are at the greatest risk for mucormycosis. Studies have shown that patients with hematologic malignancies, transplantation, neutropenia, corticosteroid therapy, and deferoxamine use are at an increased risk.^1^^,^^3^^,^^4^ Other patients at risk include those suffering burns, severe trauma, and renal failure. Diabetes mellitus, however, is the most common predisposing condition, being prevalent in 60% to 81% of patients with a diagnosis of mucormycosis.^4^^,^^5^ The accompanying state of diabetic ketoacidosis is thought to compound this risk. The persistent hyperglycemia impairs neutrophil chemotaxis and its ability to phagocytose invading fungi. In addition, the acidic environment reduces the binding of iron to transferrin, increasing free iron concentration, enhancing the conditions for fungal multiplication.^6^

Bhansali et al^7^ analyzed some of the most frequent ophthalmologic signs/symptoms associated with mucormycosis. In their retrospective review of 35 patients, ophthalmoplegia (89%), proptosis (83%), loss of vision (80%), chemosis (74%), periorbital swelling (66%), and periorbital pain (43%) were the most frequently cited complaints.^7^ These were similar in prevalence to the ophthalmic signs/symptoms reported by previous studies. When looking at nonophthalmic signs/symptoms, sinusitis, nasal discharge/ulceration, facial swelling, cranial nerve VII palsy, and palatable necrosis were the most commonly reported.^4^^,^^5^^,^^7^

**Figure 1:**
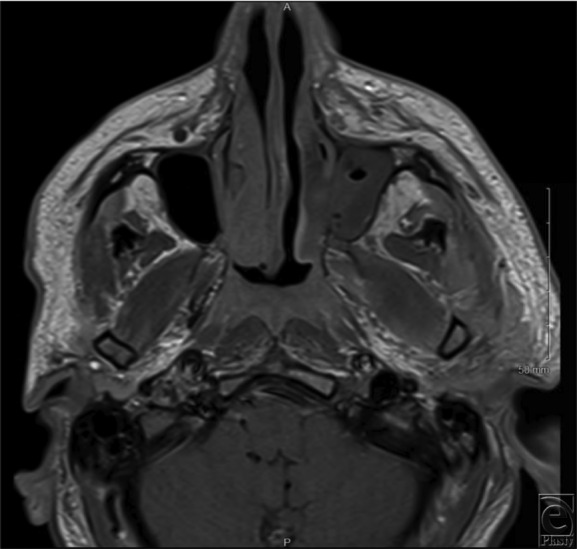
Admission T1-weighted magnetic resonance image; mucormycosis invasion along the left inferior turbinate and left maxillary sinus.

**Figure 2:**
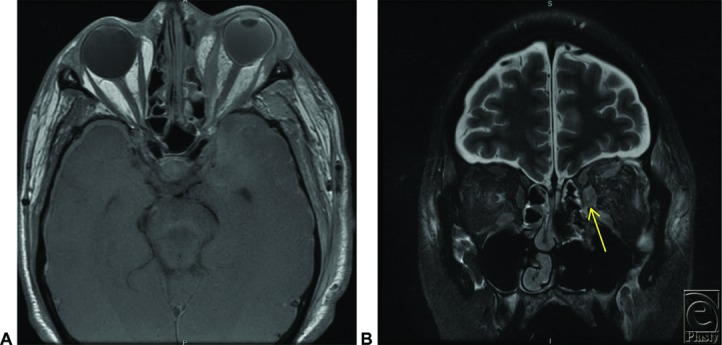
Orbital magnetic resonance image demonstrating left medial rectus involvement: (a) axial T1 SE; (b) coronal T2.

For those patients unable to report signs/symptoms secondary to altered levels of consciousness, imaging can offer an alternative means for confirming clinical suspicion. CT findings include mucosal thickening and occupation of paranasal sinuses, with the ethmoid and maxillary sinuses being more frequently involved (Fig 1).^2^^,^^7^ CT scan can also demonstrate thickening of orbital tissue, cavernous sinus thrombosis, and cranial base involvement. Magnetic resonance imaging (MRI) may show hypointense areas in both T1- and T2-weighted images (Figs 2*a* and 2*b*). Regardless of patient complaints or findings on imaging, the gold standard for diagnosis remains biopsy and histopathology.

**Figure 3:**
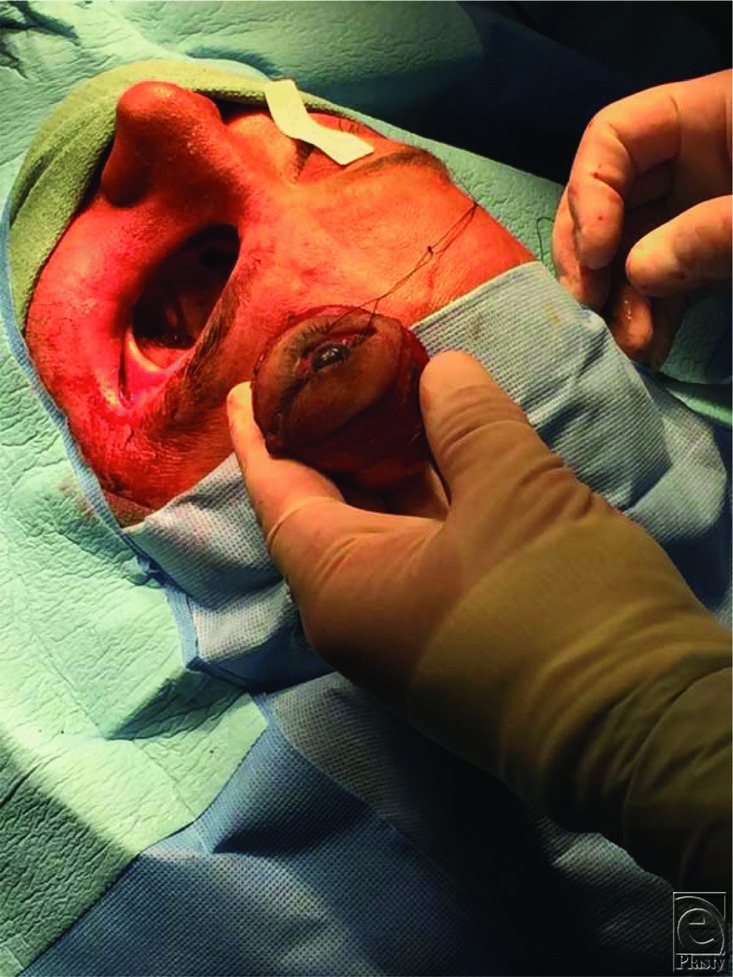
Orbital exenteration.

Those patients who have a delayed diagnosis/treatment, hemiparesis/hemiplegia, bilateral sinus involvement, leukemia, or renal disease have a lower survival rate than their counterparts.^4^ An early diagnosis, reversal of predisposing risk factors, appropriate early surgical debridement, and rapid antifungal therapy decrease morbidity and mortality.^2^ Amphotericin B has been found to be partially effective in the medical treatment of mucormycosis. Systemic administration at 1 to 1.5 mg/kg per day, or use as an intraoperative irrigant (50 mg/500 mL), has yielded improved results.^1^^-^4^,^^7^^,^^8^ Unfortunately, the vascular invasion by the fungi often precludes adequate delivery and penetration of the antibiotic into the affected areas; therefore, adjunctive surgical therapy is often warranted.^8^ A multidisciplinary approach involving various surgical specialties is utilized. Debridement may be limited to the surrounding periorbita and neurovasculature or may proceed further back toward the skull base and cranial vault, necessitating craniotomies/craniectomies, lobectomies, and even exenteration to remove the entire extent of disease (Fig 3). The combination of medical and surgical treatment has improved outcomes.^4^^,^^5^^,^^8^

Our patient was found to have mucormycosis extending from his left nare all the way to the left foramen rotundum. After turbinate and maxillary sinus debridement by otolaryngology, orbital exenteration was required, given the extent of fungal spread. A preseptal plane was taken down to the orbital rims and then transitioned to a subperiosteal plane to ensure removal of all involved orbital soft tissues. The optic nerve was divided at the apex. The infraorbital nerve was sacrificed and debrided with its surrounding maxillary soft tissues. No maxillectomy was performed. The skull base and intracranial fungal extensions were treated via intrathecal and systemic amphotericin B.
